# *Polygonatum sibiricum* Polysaccharides Protect against MPP-Induced Neurotoxicity via the Akt/mTOR and Nrf2 Pathways

**DOI:** 10.1155/2021/8843899

**Published:** 2021-01-13

**Authors:** Si Huang, Haiyan Yuan, Wenqun Li, Xinyi Liu, Xiaojie Zhang, Daxiong Xiang, Shilin Luo

**Affiliations:** ^1^Department of Pharmacy, The Second Xiangya Hospital, Central South University, Changsha 410011, China; ^2^Hunan Provincial Engineering Research Centre of Translational Medicine and Innovative Drug, Changsha 410011, China; ^3^Department of Psychiatry, The Second Xiangya Hospital, Central South University, Changsha 410011, China

## Abstract

*Polygonatum sibiricum*, a well-known life-prolonging tonic in Chinese medicine, has been widely used for nourishing nerves in the orient, but the underlying molecular mechanisms remain unclear. In this study, we found that *P. sibiricum* polysaccharides (PSP) ameliorated 1-methyl-4-phenyl-1,2.3,6-tetrahydropyridine- (MPTP-) induced locomotor activity deficiency and dopaminergic neuronal loss in an *in vivo* Parkinson's disease (PD) mouse model. Additionally, PSP pretreatment inhibited *N*-methyl-4-phenylpyridine (MPP+) induced the production of reactive oxygen species, increasing the ratio of reduced glutathione/oxidized glutathione. *In vitro* experiments showed that PSP promoted the proliferation of N2a cells in a dose-dependent manner, while exhibiting effects against oxidative stress and neuronal apoptosis elicited by MPP+. These effects were found to be associated with the activation of Akt/mTOR-mediated p70S6K and 4E-BP1 signaling pathways, as well as nuclear factor erythroid 2-related factor 2- (Nrf2-) mediated NAD(P)H quinone oxidoreductase 1 (NQO1), heme oxygenase-1 (HO-1), glutamate-cysteine ligase catalytic subunit (Gclc), and glutamate-cysteine ligase modulatory subunit (Gclm), resulting in antiapoptotic and antioxidative effects. Meanwhile, PSP exhibited no chronic toxicity in C57BJ/6 mice. Together, our results suggest that PSP can serve as a promising therapeutic candidate with neuroprotective properties in preventing PD.

## 1. Introduction

Parkinson's disease (PD) is the second most common neurodegenerative disease, with typical movement abnormalities that include resting tremor on one or both sides, rigidity, bradykinesia, and postural instability as major clinical symptoms [1]. PD is characterized by selective degeneration of dopaminergic neurons in the substantia nigra pars compacta (SNpc) and a corresponding decrease in dopaminergic innervation of the striatum; its pathological hallmark is the presence of intraneuronal ∂-synuclein aggregate inclusions known as Lewy bodies in the surviving dopaminergic neurons [2]. The etiology of PD appears to be multifactorial, involving both aging, genetic, and environmental components [3]. However, growing evidence indicates that oxidative stress is the common underlying cause associated with the profound loss of dopaminergic neurons, while several other cellular mechanisms of PD, such as aberrant protein folding, dopamine (DA) metabolism, and mitochondrial dysfunction, are all related to oxidative stress [4, 5]. Although the research on PD has been conducted for almost two centuries, a lack of treatments and validated drugs remains. Thus, there is an urgent need for promising candidates that will protect against oxidative stress and promote the proliferation of dopaminergic neurons.

Mammalian target of rapamycin (mTOR), a serine/threonine (Ser/Thr) protein kinase, is a central controller of increasing protein synthesis in response to growth and proliferation to maintain homeostasis in all cell types. mTOR pathway is initiated from growth factor receptors on the cell membrane. Activated growth factor receptors by the stimulation of their ligands trigger the activation of the phosphoinositide 3-kinase (PI3K)/Akt pathway, leading to increased mTOR activity and phosphorylated of p70 S6 kinase (p70S6K) and eukaryotic initiation factor 4E binding protein 1 (4E-BP1), resulting in increased gene transcription, cell growth, and proliferation [6]. The activation of the Akt/mTOR pathway by agents was shown to restore the activity of mTOR, p70S6K, and 4E-BP1 in mouse models of PD, which alleviated apoptosis and ∂-synuclein levels in the SN [7–9]. Thus, proper manipulation of Akt/mTOR signaling may be a potential strategy for the prevention and treatment of PD.

The brain is particularly vulnerable to oxidative stress due to its high levels of oxidizable polyunsaturated fatty acids, high oxygen consumption, and relatively low antioxidant defense capacities. Postmortem studies have demonstrated that lipid peroxidation and oxidative modification of proteins and DNA accumulate, and the levels of reduced glutathione (GSH) are decreased in SN [10–12]. Complementarily, reactive oxygen species (ROS) are generated by the imbalance of prooxidant/antioxidant homeostasis, which can trigger a cascade of events that lead to cell death [13]. However, cells have developed a complex system to defend against oxidative stress. Nuclear factor erythroid-derived 2-related factor 2 (Nrf2) is a transcription factor that regulates the expression of several antioxidant and phase II detoxifying enzymes, playing a vital role in the cellular defense mechanism of combating oxidative stress. NAD(P)H quinone oxidoreductase 1 (NQO1) and heme oxygenase 1 (HO-1) are the critical enzymes produced by the Nrf2/Keap1/ARE signaling pathway [14]. NQO1 is a member of the phase II detoxification enzyme family and catalyzes the two-electron reduction of neurotoxic dopamine (DA)-*o*-quinone that is produced by the oxidation of DA to redox-stable hydroquinone [15]. Induction of NQO1 by sulforaphane protects against neurocytotoxicity associated with DA-*o*-quinone *in vitro* and defends against 1-methyl-4-phenyl-1,2,3,6-tetrahydropyridine- (MPTP-) elicited oxidative stress *in vivo* [16, 17]. HO-1 metabolizes prooxidant heme to the antioxidant pigment biliverdin, carbon monoxide, and ferrous iron [18]. A previous study demonstrated that Nrf2 knockout mice exhibit a severe deficiency in the coordinated gene regulatory program and extreme susceptibility to oxidative damage, which suggests the irreplaceable role of Nrf2 in antioxidant defense [19]. Therefore, a strategy to stimulate the activation of the Nrf2/Keap1/ARE signaling pathway to induce the expression of NQO1 and HO-1 could combat the oxidative stress that occurs in dopaminergic neurons in PD.

As a traditional Chinese herbal medicine, *Polygonatum sibiricum* is widely distributed in most regions in the south of the Yangtze River in China. It has served as a Taoist health potion since ancient times and functions to nourish the liver and kidney and prolong life [20]. The constituents of *P. sibiricum* include polysaccharides, saponins, flavonoids, alkaloids, and a variety of trace elements, of which polysaccharides are the major pharmacologically active ingredients [21]. A recent study showed that *P. sibiricum* polysaccharides (PSP) upregulated the expression of peroxisome proliferator-activated receptor-gamma (PPAR-*γ*) and significantly improved the behavior of PD rats [22]. Treatment with the decoction prepared from *P. sibiricum* might also activate the telomerase activity in the brain and gonadal tissues of aging animals, supporting the potential role for PSP in the treatment of PD [23]. However, to date, no study has determined the underlying pharmacological mechanisms of PSP.

In this study, by using MPTP treatment as an *in vivo* PD model, we evaluated the ability of PSP to protect against MPTP-induced motor deficits and dopaminergic neuron loss. Based on the N2a cell culture model, we confirmed that the possible mechanisms underlying its effects are associated with Akt/mTOR-mediated antiapoptotic and Nrf2-regulated antioxidative effects. PSP exhibited no chronic toxicity in C57BL/6 mice. Our results suggest that PSP may serve as a promising therapeutic agent for PD.

## 2. Materials and Methods

### 2.1. Extraction of Polysaccharides from *P. sibiricum*

The rhizomes of *P. sibiricum* were purchased from Yi-Pu-Yuan Huangji Technology Co., Ltd., Xinhua, Hunan province, China, in June of 2017 and were authenticated by Dr. Gao Liu (Hunan Academy of Chinese Medicine). A voucher specimen (No. 20170611) was deposited in the Hunan Provincial Engineering Research Centre of Translational Medicine and Innovative Drug, Changsha, China. The extraction of polysaccharides was performed by a previously described procedure [24]. In brief, the dried crude powder of the rhizomes of *P. sibiricum* (1.5 kg) was defatted with 95% EtOH for 3 days and then extracted with boiling water (1 : 10, v/v) for three cycles, 3 hr each time. The combined extracts were pooled and concentrated; then, three volumes of 95% EtOH were added slowly by stirring to precipitate the polysaccharides, and kept at 4°C overnight. The polysaccharide pellets were obtained by centrifugation and repeatedly washed with possibly less amount of ethanol. The refined polysaccharide pellets were completely dissolved in an appropriate volume of water and then deproteinated with Sevag reagent (CHCl_3_ : n − BuOH = 4 : 1, v/v) for 30 min under the magnetic force stirring, and the procedure was repeated two times. Finally, the extracts were centrifuged to remove the insoluble substance, and the supernatant was lyophilized in a freezer dryer to give the polysaccharides a brown fluffy shape.

### 2.2. PSP Treatment, MPTP Injection, and Behavioral Test

**Figure 1:**
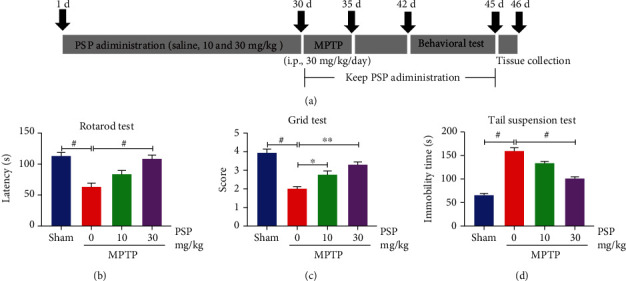
PSP rescues MPTP-induced motor dysfunction in mice. (a) The schedule of a combined protocol of PSP and MPTP, as well as behavioral evaluations. Mice underwent behavioral tests: rotarod test (b), grid test (c), and tail suspension test (d) were measured. Data represent the mean ± S.E.M (*n* = 7 or 8). *p* < 0.05, ^∗∗^*p* < 0.01, and ^#^*p* < 0.001 by one-way ANOVA with Tukey's multicomparisons test.

C57BL/6J male mice (8 weeks old, 22-25 g) were purchased from Hunan SJA Laboratory Animal Co., Ltd., Changsha, China, which were housed in SPF feeding conditions. PSP was dissolved in sterile water. Two batches of mice were performed in the animal experiment. For the firth batch of mice, after one week of adaptation, the mice were randomly divided into the sham group (*n* = 8), experimental groups (vehicle, 10 mg/kg (body weight), and 30 mg/kg PSP, *n* = 8 for each group). PSP was administrated by oral gavage once daily for 4 weeks, and then, the mice in experimental groups were injected a daily of MPTP (i.p., 30 mg/kg) for five consecutive days. Motor impairments were tested one week after MPTP treatment with rotarod tests, grid tests, and tail suspension tests. In the rotarod tests, mice were trained for 2 min at a speed of 4 rpm and then performed eight trials for a maximum of 5 min with increasing speed starting from 4 rpm to 40 rpm. The fall-off time was recorded. For inverted grid tests, mice were placed in the center of a screen (30 × 30 cm) with a 1 cm wide mesh. The screen was inverted head-over-tail and placed on supports 40 cm above an open cage with deep bedding. Mice were timed until they released their grip or remained for 60 s. In terms of the tail suspension test, mice were suspended to a bar by fastening their tails. The distance between the mouse's nose and the apparatus was kept to 25 cm. The hanging session of each mouse was recorded by a video for 6 min, and the agitation and immobility times of each mouse during the test were analyzed. Maintain PSP daily administration during MPTP injection and behavioral testing until brain tissues were obtained. The total experimental period was summarized in Figure 1(a). The second batch of mice (*n* = 8 for each group) did not conduct behavioral tests after the same treatment of PSP and MPTP, and the brain tissues were collected for the next researches. The animal experiments were carried out following the Guiding Principles of Animal Ethics Committee of the Second Xiangya Hospital, Central South University, Changsha, China.

### 2.3. Immunostaining

For immunofluorescence staining, cells on slides were fixed with 4% paraformaldehyde for 10 min, then washed with PBS for three times and blocked in blocking buffer (1% BSA, 0.3% Triton X-100, and 10% goat serum in PBS) for 1 h, followed by the incubation with anti-TH at 4°C for overnight. After washing with carrier buffer (1% BSA, 0.3% Triton X-100, and 1% goat serum in PBS), they were incubated with Alexa Fluor 555-conjugated anti-rabbit IgG (#A0453, Beyotime) for 2 h at room temperature, followed by DAPI staining for 5 min. Then, cell slides were mounted for taking pictures. Immunochemistry staining was performed according to the manufacturer's protocol (#95-6143, Invitrogen). In brief, free-floating 25 *μ*m-thick serial sections were tightly attached to the microslides and then were treated with 0.3% hydrogen peroxide for 10 min followed by incubation with anti-TH at 4°C for overnight. After washing with PBS, the sections were incubated with a biotinylated second antibody (Reagent 1B) followed by the conjugate enzyme (Reagent 2) for each 10 min. Finally, a chromogen AEC single solution was used to develop the signals, and pictures were captured on a microscope (BX51TF, Olympus, Tokyo, Japan).

### 2.4. Stereological Quantification of TH-Positive Cells

The number of TH-positive cells in the SN was estimated by random sampling stereo counting. For each animal, every fifth section throughout the rostrocaudal level of the SN and every fifth section covering the entire level of the striatum were incorporated into the counting procedure. The researcher was blinded to the conditions of the experiment.

### 2.5. Dopamine Determination

Dopamine levels were determined by HPLC with fluorometric detection as previously reported [25]. In brief, substantia nigra and striatum samples were homogenized in 0.2 M perchloric acid (HClO4) containing 3 mM cysteine and then centrifuged at 13,000 × g for 15 min at 4°C. Aliquots of supernatant fractions were filtered with 0.2 *μ*m HT Tuffryn membrane, then analyzed in HPLC using a reverse-phase column (C18) with acetate buffer (12 mM, pH 4.0)-methanol (86 : 14, v/v) as mobile phase. The flow rate was 1 mL/min, and the fluorescence measurements were carried out at 320 nm with excitation at 279 nm. The dopamine content (ng/sample) was then quantified by comparison to internal standards, with a standard curve generated with 0.1-5 ng of dopamine standard. The protein level (mg/sample) was determined with Bradford assay with a standard curve generated with 0-10 *μ*g bovine serum albumin.

### 2.6. GSH/GSSG Analysis

GSH/GSSG Ratio Detection Assay Kit (Fluorometric-Green, #ab138881, Abcam) was used to determine the GSH/GSSG ratio in cells or brain tissues according to the manufacturer's protocol. In brief, the whole-cell lysates or brain substantia nigra lysates were diluted to 1 : 80 for GSH analysis, and a series dilution of GSH and GSSG stock standards were prepared as standards. A one-step fluorometric reaction of samples with respective assay buffer and probes was incubated for 1 h protected from light at room temperature. Then, fluorescence intensity was monitored at EX/EM of 490/520 nm. GSH was calculated from the standard curve, and GSSG = (total glutathione − GSH)/2.

### 2.7. Western Blotting

Western blotting was performed using a standard protocol. Cells or brain tissues were sonicated and lysed with RIPA lysis buffer, and insoluble pellets were removed by centrifugation at 15,000 × g for 15 min at 4°C. Protein concentration was measured by Bradford assay (#PQ0041, MultiSciences Biotech Co., Ltd.), and the extracts were stored at -80°C until analysis. Equal amount of protein (20-40 *μ*g) was loaded for blotting with anti-p-Akt/Akt (#4060/#4691, Cell Signaling Technology), p-mTOR/mTOR (#5536/#2983, Cell Signaling Technology), p-p70S6K/p70S6K (#9204/#9202, Cell Signaling Technology), p-4E-BP1/4E-BP1 (#2855/#9644, Cell Signaling Technology), Nrf2 (#12721, Cell Signaling Technology), NQO1 (#sc-32793, Santa Cruz Biotechnology), Cleaved Caspase-3 (#9661, Cell Signaling Technology), TH (#58844, Cell Signaling Technology), HO-1 (#sc-390991, Santa Cruz Biotechnology), Gclc (#ab190685, Abcam), Gclm (#ab126704, Abcam), and anti-*β*-actin (#ab8227, Abcam).

### 2.8. ROS Staining

Cells were plated onto slides to culture overnight and treated with different concentrations of PSP (0, 100, 200 *μ*g/mL) and MPP+ at different points in time, then fixed with 4% paraformaldehyde for 10 min. Cells were firstly washed twice with Hank's balanced salt solution containing Ca^2+^/Mg^2+^, and then added with 5 *μ*M CM-H_2_DCFDA (ROS dye, #C6827, Invitrogen) that was diluted in PBS for 1 h at 37°C. Then, washed the slides three times with PBS and allowed them to recover for 10 min at 37°C. ROS species contain superoxide anion, hydrogen peroxide, and hydroxyl radical were reacted with CM-H_2_DCFDA. The images were captured by a confocal microscope (FV3000, Olympus, Tokyo, Japan).

### 2.9. Cell Culture and CCK-8 Assay

N2a cells were cultured and maintained in Dulbecco's Minimal Essential Medium (DMEM) supplemented with 10% fetal bovine serum (FBS), 1% penicillin/streptomycin at 37°C, and 5% CO_2_. The cell proliferation rate was evaluated in triplicate by using Cell Counting Kit-8 (CCK-8, #6005, NCB Biotech) according to the manufacturer's protocol. In brief, N2a cells were cultured in 96-well plates overnight and were treated with PSP with 0, 1, 10, 100, 200, and 400 *μ*g/mL in various wells. After incubation for 1 day and 3 days, 10 *μ*L CCK-8 was added to each well and continued to culture for 1 h in the incubator. The optical density (OD) was determined at 450 nm using a microplate reader (#infinite F50, TECAN). The CCK-8 assay was repeated three times.

### 2.10. Hematoxylin and Eosin Staining

The brain, liver, and kidney of mice were collected and immediately fixed with 4% formaldehyde. After immersion, these organs were dehydrated by gradual soaking in alcohol and xylene, embedded in paraffin, and then sliced into 5 *μ*m-thick sections. The sections were stained with standard hematoxylin and eosin (H&E) staining protocol [26]. The sections were visualized under a microscope (BX51TF, Olympus, Tokyo, Japan).

### 2.11. MAO-B Activity Assay

Amplex Red Hydrogen Peroxide/Peroxidase Assay Kit (#A22188, Sigma) was used to determine the enzymatic activity of MAO-B. In brief, cell or SN lysates (15 *μ*g) were incubated with 100 *μ*L working solution (400 *μ*M Amplex Red Reagent, 2 U/mL horseradish peroxidase, 2 mM p-tyramine, and MAO-A-specific inhibitor clorgyline) at 37°C for 1 h; then, the fluorescence of MAO-B activity was measured in a fluorescence plate reader using excitation at 570 nm and emission at 585 nm.

### 2.12. Statistical Analysis

All data were expressed as means ± S.E.M. from three or more independent experiments. Statistical analysis was performed with Prism 7.0 (GraphPad Software). Histological data were analyzed by Student *t*-test or one-way ANOVA. The threshold for significance for all experiments was set ^∗^*p* < 0.05, and smaller *p* values were represented as ^∗∗^*p* < 0.01 and ^#^*p* < 0.001.

## 3. Results

### 3.1. PSP Rescue MPTP-Induced Motor Dysfunction in Mice

PSP has neuroprotective effects on cerebral ischemia-reperfusion injury in rats [27]. To verify the *in vivo* roles of PSP in PD, we administered PSP (10 mg/kg and 30 mg/kg) by oral gavage daily to 2-month-old male mice for 30 days followed by treatment with MPTP (experimental groups, i.p. 30 mg/kg) or saline (sham group) for five consecutive days. The mice were evaluated for motor dysfunction during the last 3 days of PSP administration. The study design is depicted in Figure 1(a). The rotarod test showed that MPTP led to significant motor disorder compared to the sham group, which was ameliorated upon treatment with 30 mg/kg PSP (Figure 1(b)). Similar observations were made in both the grid test and tail suspension test. Motor improvement occurred with the administration of 10 mg/kg PSP and was more pronounced in the 30 mg/kg group (Figures 1(c) and 1(d)). These results suggest that PSP can ameliorate PD-related motor deficits caused by MPTP.

### 3.2. PSP Attenuates MPTP-Induced Dopaminergic Neurodegeneration *In Vivo*

**Figure 2:**
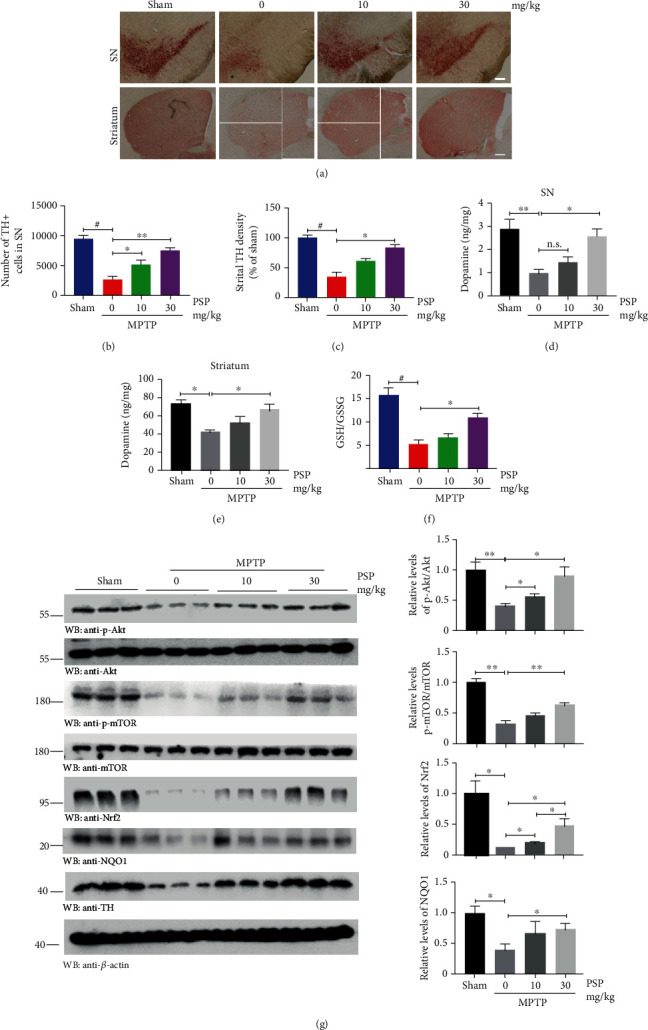
PSP attenuates MPTP-induced dopaminergic neurodegeneration *in vivo*. (a) PSP displayed excellent neuroprotection for dopaminergic neurons. TH immunostaining of the substantia nigra (SN) and striatum of mice with indicated PSP and MPTP treatment. Scale bar, 200 *μ*m. Unbiased stereological cell counts in the SN (b) and total density of striatal dopaminergic terminals (c). Data represent the mean ± S.E.M (*n* = 6 mice for each group). (d), (e) Dopamine concentrations in SN and striatum were increased by PSP pretreatment. (f) GSH/GSSG ratio analysis for SN lysates showed that oxidative stress was weakened by PSP. Data represent the mean ± S.E.M (*n* = 3). (g) Western blot analyses for SN lysates with various indicated antibodies. Blot data are representative of three independent experiments. Quantifications of relative protein levels are shown in the right panel (*n* = 3). *p* < 0.05, ^∗∗^*p* < 0.01, and ^#^*p* < 0.001 by Student's *t*-test (WB statistics) or one-way ANOVA with Tukey's multicomparisons test (b)–(f).

Because one of the crucial pathological changes in PD is a loss of dopaminergic neurons (tyrosine hydroxylase- (TH-) positive neurons), we conducted immunohistochemical (IHC) staining to detect the integrated intensity of TH expression in the SN and striatum. The results showed that dopaminergic neurons in the SN and its projection in the striatum were substantially diminished by MPTP in the vehicle-treated mice as compared to the sham group. Again, 10 mg/kg PSP attenuated the degeneration of dopaminergic neurons, and strong neuroprotective effects occurred up to 30 mg/kg (Figure 2(a)), which were confirmed by the quantitative analysis (Figures 2(b) and 2(c)). High-performance liquid chromatography with fluorometric detection was used to detect the DA concentrations in the SN and striatum, and the results were consistent with the TH staining (Figures 2(d) and 2(e)). Because MPTP is metabolized into MPP+ *in vivo*, it specifically causes oxidative stress in dopaminergic neurons after crossing the blood-brain barrier. As one of the important indicators, GSH/GSSG ratio analysis for SN demonstrated that 30 mg/kg PSP led to the lowest oxidative stress among the experimental groups, provoked by MPTP treatment, in alignment with its prominent neuroprotective activity (Figure 2(f)). To determine the potential protein expression changes in the SN, the immunoblotting analysis was performed. Interestingly, p-Akt and p-mTOR, two critical proteins involved in cell proliferation, were significantly downregulated in the MPTP group with no PSP treatment but were restored after PSP administration, especially at 30 mg/kg improving expression by 2.2-fold and 2.0-fold, respectively. Similarly, the same patterns were observed for Nrf2 and NQO1, which participate in antioxidant stress. As expected, the expression of TH was consistent with IHC staining (Figure 2(g)). These data show that PSP attenuates MPTP-induced dopaminergic neurodegeneration, and the Akt/mTOR and Nrf2 pathways might be activated in this process.

### 3.3. PSP Inhibits MPP+-Induced Neuronal Apoptosis by Restoring the Akt/mTOR Pathway *In Vitro*

**Figure 3:**
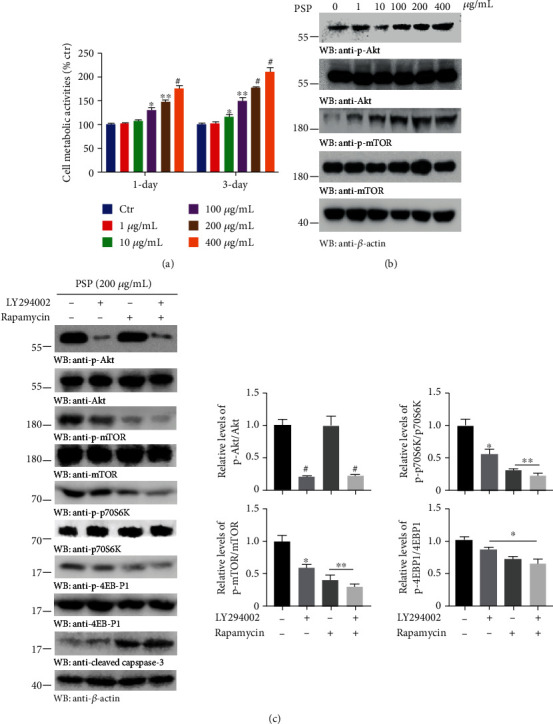
PSP inhibits MPP+-induced neuronal apoptosis by restoring the Akt/mTOR pathway. (a) Effect of PSP on the proliferation of N2a cells. The cells were treated with indicated concentrations of PSP for 1 d or 3 d, and cell viability was determined by CCK-8 assay (*n* = 3). (b) Western-blot analyses showed that PSP upregulated the expression of p-Akt and p-mTOR. The N2a cells were treated with PSP for 24 h. (c) The phosphorylation of Akt, mTOR, p70S6K, and p-4E-BP1 by the stimulation of PSP were blocked after the pretreatment of inhibitors LY294002 (10 *μ*M) and rapamycin (250 nM) alone or in combination. Quantification of relative protein levels is shown in the right panel (*n* = 3). *p* < 0.05, ^∗∗^*p* < 0.01, and ^#^*p* < 0.001 by Student's *t*-test.

Disturbances in the balance of the Akt/mTOR signaling pathway in the brain can impair neuronal functions and have detrimental consequences on neuronal regeneration after damage [28]. To test the hypothesis that PSP prevents MPP+-induced neuronal apoptosis by activating the Akt/mTOR pathway, suggested by our *in vivo* western blot results, we first examined the proliferative potential of PSP on mouse brain neuroma cells Neuro-2a (N2a cells). The Cell Counting Kit-8 (CCK-8) assay was carried out to evaluate the viability after the cells were treated with an increasing concentration of PSP for 24 h and 72 h. The results demonstrated that PSP dose-dependently increased cell proliferation after the concentration reached 100 *μ*g/mL for 24 h or 10 *μ*g/mL for 72 h, with the corresponding cell metabolic activity increasing by up to 174% and 209% after 400 *μ*g/mL stimulation, respectively (Figure 3(a)). Further, the western blot analysis revealed that both Akt and mTOR phosphorylation was upregulated in a dose-dependent manner when the N2a cells were treated with PSP for 24 h (Figure 3(b)). The phosphorylated Akt and mTOR levels with PSP treatment were inhibited by the effective and selective inhibitors LY294002 and rapamycin, respectively, which resulted in the inactivation of mTOR-mediated p-p70S6K and p-4E-BP1, followed by an increase in expression of cleaved caspase-3 (Figure 3(c)). These data suggest that PSP inhibits MPP+-induced neuronal apoptosis by restoring the Akt/mTOR pathway.

### 3.4. PSP Rescues MPP+-Induced Oxidative Stress Damage by Activating the Nrf2 Pathway In Vitro

**Figure 4:**
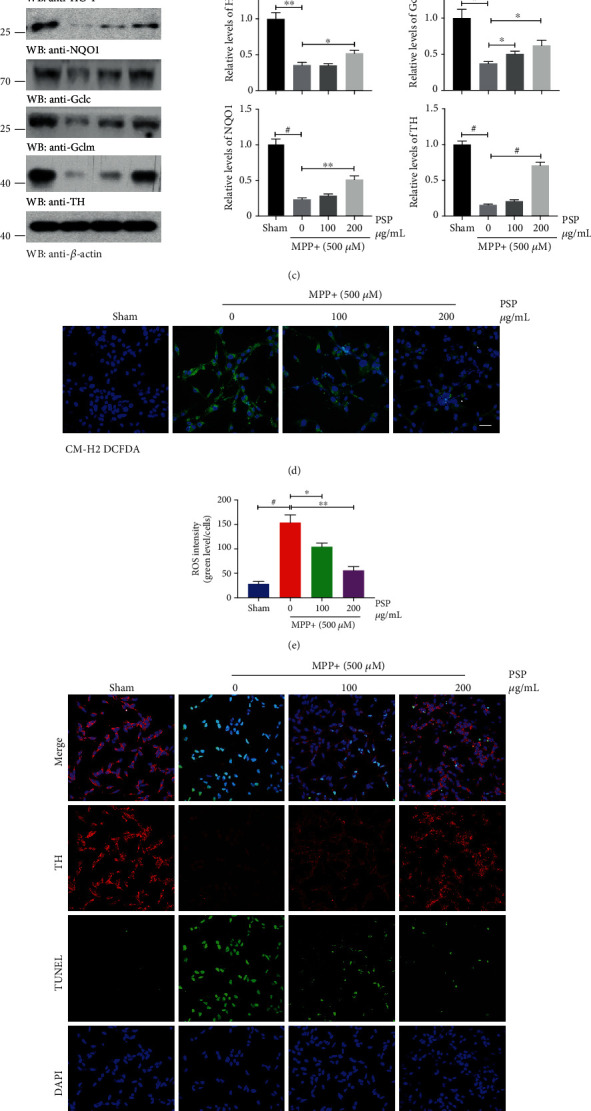
PSP rescues MPP+-induced oxidative stress damage by activating the Nrf2 pathway. (a) LDH assay showed PSP exerts strong cytoprotective activity. The medium was collected from N2a cells that were pretreated with PSP with indicated concentrations for 24 h then MPP+ (500 *μ*M) treatment overnight before cell harvesting (*n* = 3). (b) GSH/GSSG ratio analysis for whole-cell lysates of N2a cells (*n* = 3). (c) Western blot analyses showed that the expression of the proteins in the Nrf2 signaling pathway, as well as TH, was restored after PSP treatment. Blot data are representative of three independent experiments. Quantification of relative protein levels is shown in the right panel (*n* = 3). (d) Representative images of ROS staining by the indicator dye CM-H_2_DCFAD for N2a cells. Scale bar, 50 *μ*m. (e) Quantification of ROS intensities using the ratio of fluorescent intensity and cell numbers (*n* = 5). (f) Representative images of immunofluorescent staining of TH/TUNEL in N2a cells treated with indicated conditions. Scale bar, 50 *μ*m. Quantification of TH intensity (g) and TUNEL-positive cells (h) are shown (*n* = 5). Data represent the mean ± S.E.M. *p* < 0.05, ^∗∗^*p* < 0.01, and ^#^*p* < 0.001 by Student's *t*-test (WB statistics) or one-way ANOVA with Tukey's multicomparison test (e), (g), and (h).

Our *in vivo* results prompted us to examine the antioxidant potential of PSP with a MPP+-induced oxidative damage cell model *in vitro*. In the experimental groups, N2a cells were first pretreated with PSP at the indicated concentrations for 24 h, followed by incubation with MPP+ (500 *μ*M) overnight. The medium was collected and monitored with a LDH assay. Remarkably, MPP+-elicited cell death was significantly repressed by PSP, especially for 200 *μ*g/mL (Figure 4(a)). In addition, MPP+ also resulted in a consistent, significant decrease in the GSH/GSSG ratio, which was restored by PSP predose (Figure 4(b)). Immunoblot analysis showed that N2a cells treated with 200 *μ*g/mL PSP significantly upregulated the expression of Nrf2 and its downstream antioxidant proteins and detoxifying enzymes, HO-1, NQO, and glutamate-cysteine ligase modulatory subunit (Gclm), as well as the GCL catalytic subunit (Gclc). Meanwhile, as a marker of dopaminergic neurons, the changes in TH expression also revealed a similar pattern (Figure 4(c)). ROS fluorescent probe staining indicated PSP had an obvious protective effect on the oxidative status, and the quantitative analysis of ROS intensities mirrored the discoveries (Figures 4(d) and 4(e)). To further assess the cytoprotective effects, we performed a costaining of TUNEL and TH in N2a cells treated with PSP and MPP+ and found that PSP exhibited strong cytoprotective activity, which led to the weak TUNEL signals but strong TH activity (Figures 4(f)–4(h)). Thus, our finding supports the notion that PSP rescues MPP+-induced oxidative damage by activating the Nrf2 pathway.

### 3.5. Oral Administration of PSP Presents No Toxicity for Mice

**Figure 5:**
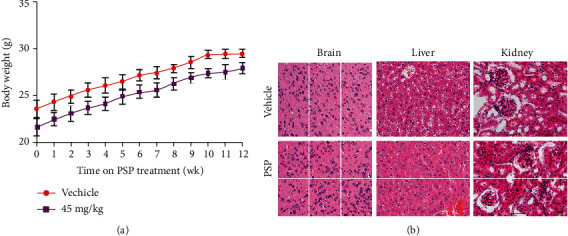
Oral administration of PSP presents no toxicity for mice. (a) The monitoring curve of body weight demonstrated no significant difference between the vehicle (sterile water) and PSP treatment group. (b) Representative images of H&E staining of the brain, liver, and kidney. C57BJ/6 mice regardless of gender (20-25 g) were treated with a daily dose of 45 mg/kg of PSP for 12 consecutive weeks (*n* = 4). Continuous weekly weight records were kept, and the brain, liver, and kidney were collected for pathological analysis at the end of the study. Scale bar, 40 *μ*m.

Traditional Chinese medicine may require to be taken for a long time; the safety of PSP had been taken into consideration. We performed a 12-week administration with a daily dose of 45 mg/kg PSP to evaluate the chronic toxicity in C57BJ/6 mice regardless of gender (20-25 g). Biochemical analyses of mouse blood (RBC, HB, WBC, and ESR) showed that they were in the normal ranges (data not shown). Consecutive weekly weight records showed healthy growth in mice (Figure 5(a)), and the H&E staining of tissue sections from the brain, liver, and kidney suggested that there was no significant difference between mice administered PSP and those dosed sterile water (Figure 5(b)).

Thus, the study indicates that the long-term oral administration of PSP presents no chronic toxicity for mice.

## 4. Discussion

Although dopaminergic drugs such as DA prodrug (levodopa), levodopa synergists, DA receptor agonists, and DA release promoters have become the first-line drugs for clinical treatment of PD; they are all based on therapy after the DA neurons have degenerated. It is crucial to find promising drugs to prevent the loss of DA neurons during the development of PD. Our findings showed that the reasons for PSP to improve parkinsonian behaviors are because PSP not only promotes dopaminergic neuron proliferation but also protects them against oxidative stress injury, which provides potential protective effects in the early stages to combat the PD development. The rhizome extract of *P*. *sibiricum* scavenge 1,1-diphenyl-2-picryl-hydrazyl (DPPH) and hydroxyl radicals and decrease ROS levels in liver cells [29]. The DPPH radical scavenging activity assay showed that PSP with microwave-assisted degradation exhibited stronger antioxidant activity than the original neutral polysaccharides [30]. Purified polysaccharides from *P*. *sibiricum* had protective effects against Aß_25-35_-induced apoptosis in PC12 cells, and the mechanism was associated with the enhancement of the PI3K/Akt signaling pathway [31]. These reports, together with our findings, suggest that PSP has the potential to treat PD.

The Akt/mTOR pathway is involved in regulating neuronal differentiation and survival, as well as learning and memory, synaptic plasticity, and neuronal oxidative stress [32–34]. In the Akt/mTOR signaling network of the nervous system, the activation of Akt is initiated from the binding of a variety of neurotrophic factors to their specific membrane receptors. mTOR is a downstream molecule of Akt and can be activated by phosphorylated Akt, which further regulates the phosphorylation of its best-characterized downstream effectors, namely, p70S6K and 4E-BP1 [35]. Studies have demonstrated that neurotoxins, such as MPP+, 6-hydroxydopamine, and rotenone, repress the phosphorylation of Akt, mTOR, p70S6K, and 4E-BP1 and that loss of Akt leads to neuronal loss and converge on caspase-3 activation, resulting in nuclear degradation and cellular morphological change [28, 36]. Consistent with these reports, we found that pretreatment with PSP strongly reversed the MPTP/MPP+-inhibited phosphorylation of Akt and mTOR (Figure 2(g)), whereas these effects were partially reduced after the inhibition of Akt and mTOR alone, and their combined inhibition caused a more potent suppression, either the activation of p70S60K and 4E-BP1 or the neuronal survival detected by cleaved-caspase 3 (Figure 3(c)). Although the kinase mTOR is a major negative regulator of autophagy and the activation of autophagy may promote the clearance of ∂-synuclein, the interplay between mTOR and autophagy is complex in PD [37]. One report showed that though the neuroprotection of sulforaphane is linked to the alterations of mTOR signaling, it does not seem mutually dependent on autophagy processes [28]. Thus, we suspect that PSP may perform a similar underlying mechanism, and the levels of autophagy and ∂-synuclein aggregates are worth exploring in the A53T transgenic mice.

Oxidative stress is defined as an imbalance between the production of ROS and cellular antioxidant activity. Dopaminergic neurons are particularly prone to oxidative stress due to the presence of an abundance of ROS-generating enzymes, such as TH and monoamine oxidase, in the synthesis and metabolism of dopamine. Typically, DA oxidizes to DA-*o*-quinone and can form adducts with mitochondrial complex I, III, and V inducing mitochondrial dysfunction [38]. It also can undergo intramolecular cyclization to aminochrome, which is one-electron reduced by flavoenzymes to leukoaminochrome-*o*-semiquinone radical, and further autooxidizes immediately to aminochrome-generating oxidative stress [39]. In addition, the nigral dopaminergic neurons contain iron. Dopamine forms complex with ferric iron (Fe^3+^) that is taken up into the neurons by DA transporters to catalyze through Fenton serial reactions, in which hydrogen peroxide (H_2_O_2_) and hydroxyl radical can contribute to further oxidative stress [39]. Meanwhile, exposure to oxidizing environments, such as MPTP treatment, can accelerate these reaction processes. Thus, oxidative stress is regarded to be the common underlying mechanism leading to the dysfunction and demise of dopaminergic neurons, even if there are mutations, such as ∂-synuclein, PINK, and Parkin, which occur in genetic cases of PD. Our study found that PSP could decrease the ROS levels *in vitro* (Figures 4(d) and 4(e)) and increase DA content and augment GSH/GSSG ratio *in vivo* (Figures 2(d)–2(f)), which indicated PSP may resist the intrinsic oxidative stress induced by dopamine to protect neurons from demise.

Previous studies have shown that Akt is one of the potential upstream signaling regulators to Nrf2, and the phosphorylation of Akt could augment the nuclear translocation of Nrf2 through PI3K/Akt/GSK-3*β* signaling events, which triggered the expression of a series of antioxidant proteins and detoxifying enzymes [40, 41]. Thus, the Akt/Nrf2-NQO1/HO-1/Gcls pathway provides rapid feedback for the antioxidant defense system. Here, we reported that the underlying mechanisms of PSP attenuating MPP+-induced neurotoxicity were involved in Nrf2 and Akt/mTOR pathways. There are reasons to speculate that the phosphorylation of Akt is the center of coupling these two signaling pathways, which facilitate PSP has a dual function that promotes the dopaminergic neuron proliferation and protects them against oxidative stress damage.

MPTP is lipid-soluble, readily penetrates the blood-brain barrier (BBB), and enters the brain cells. MPTP was converted to MPP+ by monoamine oxidase B (MAO-B) that is stored in astrocytes and serotonergic neurons. The toxic MPP+ reaches the extracellular fluid and then is transported by the DA transporter in DA nerve terminals and further uptake by mitochondrial to interfere with mitochondrial respiration [42]. To verify if PSP affects the conversion of MPTP to MPP+ *in vivo* and thus reduces MPTP-induced DA toxicity, we performed the MAO-B activity assay for N2a cells and SN lysate after PSP treatment. The results showed MAO-B-specific inhibitor pargyline strongly inhibited MAO-B activity, but PSP cannot play the same role (Figure S1A), as well as the equal enzyme activities of SN between vehicle and PSP treatment (45 mg/kg) (Figure S1B), suggesting the underlying mechanism of the *in vivo* neuroprotection of PSP is by activating the intracellular signaling pathways rather than hinder MPTP metabolism.

Despite the considerable therapeutic potential of PSP in PD, it is difficult to define which compound in PSP regulates these protective effects. Recently, phytochemical studies have revealed that several purified polysaccharides, such as a highly branched galactomannan named PSW-1a and two-branched homogalactans named PSW-1b-2 and PS-WNP, were isolated from PSP [31, 43]. Based on our current research, a single component isolated in PSP will provide more insights into the pharmacological mechanism for its protective functions on dopaminergic neurons. Thus, our future investigation will be aimed at analyzing the polysaccharide components in PSP and then precisely pointing out which polysaccharides are essential for benefiting dopaminergic neurons.

## 5. Conclusion

In summary, our study demonstrated that polysaccharides extracted from the rhizome of *Polygonatum sibiricum* appeared to improve the parkinsonian behaviors and protect dopaminergic neurons against demise in the classical mice model of MPTP-induced PD. The mechanism investigation revealed that Akt/mTOR-mediated antiapoptotic and Nrf2-regulated antioxidative pathways were involved in the neuroprotective effect of PSP. In short, PSP can be considered a promising candidate for PD, and further detailed researches are needed.

## Data Availability

All data supporting the conclusions of this article are included in this article.

## Supplementary Materials

Supplementary Materials MAO-B activity assay for N2a cells and SN lysate after PSP treatment was detected in the Varioskan Flash Multimode Reader (Thermo Scientific) with excitation at 570 nm and emission at 585 nm. Figure S1. PSP does not affect the MAO-B enzyme activity. (A) N2a cells were treated with vehicle, PSP (400 *μ*g/mL), and pargyline (5 *μ*M) for 24 h, and then, the cell lysates were subjected to MAO-B activity assay. (B) No significant difference in MAO-B activity between vehicle- and PSP-treated SN. The data represent the mean ± S.E.M (*n* = 3) and were analyzed by Student *t*-test.
