# Supplementary figures and images for: Polygonatum sibiricum Polysaccharides Protect against MPP-Induced Neurotoxicity via the Akt/mTOR and Nrf2 Pathways
Source: Oxid Med Cell Longev. 2021 Jan 13;2021:8843899. doi: 10.1155/2021/8843899 (PMC7817274; doi:10.1155/2021/8843899)

**Figure S1**

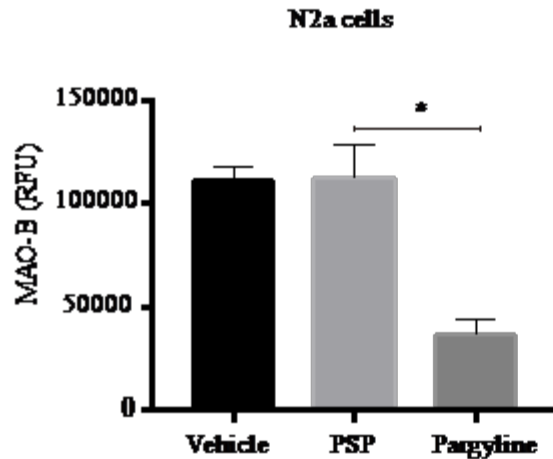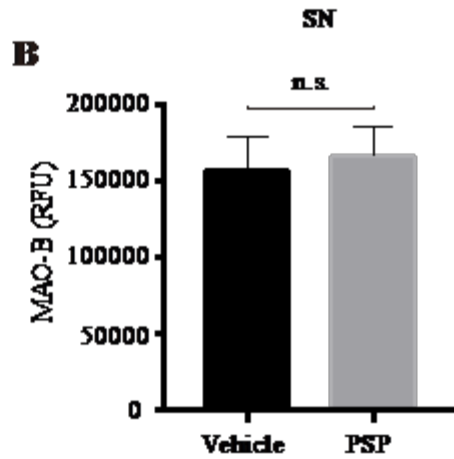

Supplement: Supplementary Materials — MAO-B activity assay for N2a cells and SN lysate after PSP treatment was detected in the Varioskan Flash Multimode Reader (Thermo Scientific) with excitation at 570 nm and emission at 585 nm. Figure S1. PSP does not affect the MAO-B enzyme activity. (A) N2a cells were treated with vehicle, PSP (400 μg/mL), and pargyline (5 μM) for 24 h, and then, the cell lysates were subjected to MAO-B activity assay. (B) No significant difference in MAO-B activity between vehicle- and PSP-treated SN. The data represent the mean ± S.E.M (n = 3) and were analyzed by Student t-test. [file 8843899.f1.pdf]
